# New insights into biomarkers and risk stratification to predict hepatocellular cancer

**DOI:** 10.1186/s10020-025-01194-6

**Published:** 2025-04-23

**Authors:** Katrina Li, Brandon Mathew, Ethan Saldanha, Puja Ghosh, Adrian R. Krainer, Srinivasan Dasarathy, Hai Huang, Xiyan Xiang, Lopa Mishra

**Affiliations:** 1 The Institute for Bioelectronic Medicine, Feinstein Institutes for Medical Research & Cold Spring Harbor Laboratory, Department of Medicine, Division of Gastroenterology and Hepatology, Northwell Health, NY, 11030 USA; 2 Cold Spring Harbor Laboratory, Cold Spring Harbor, NY, 11724 USA; 3 Division of Gastroenterology and Hepatology, Cleveland Clinic, Cleveland, OH 44106 USA; 4 Center for Immunology and Inflammation, Feinstein Institutes for Medical Research, Donald and Barbara Zucker School of Medicine at Hofstra, Northwell Health, Manhasset, NY 11030 USA; 5 Department of Surgery, George Washington University, Washington, DC 20037 USA

**Keywords:** Liver cancer, Cirrhosis, Biomarker, Early diagnosis, Risk stratify

## Abstract

Hepatocellular carcinoma (HCC) is the third major cause of cancer death worldwide, with more than a doubling of incidence over the past two decades in the United States. Yet, the survival rate remains less than 20%, often due to late diagnosis at advanced stages. Current HCC screening approaches are serum alpha-fetoprotein (AFP) testing and ultrasound (US) of cirrhotic patients. However, these remain suboptimal, particularly in the setting of underlying obesity and metabolic dysfunction-associated steatotic liver disease/steatohepatitis (MASLD/MASH), which are also rising in incidence. Therefore, there is an urgent need for novel biomarkers that can stratify risk and predict early diagnosis of HCC, which is curable. Advances in liver cancer biology, multi-omics technologies, artificial intelligence, and precision algorithms have facilitated the development of promising candidates, with several emerging from completed phase 2 and 3 clinical trials. This review highlights the performance of these novel biomarkers and algorithms from a mechanistic perspective and provides new insight into how pathological processes can be detected through blood-based biomarkers. Through human studies compiled with animal models and mechanistic insight in pathways such as the TGF-β pathway, the biological progression from chronic liver disease to cirrhosis and HCC can be delineated. This integrated approach with new biomarkers merit further validation to refine HCC screening and improve early detection and risk stratification.

## Background

Worldwide, nearly 800,000 deaths from liver cancer were reported in 2022, mostly from hepatocellular carcinoma (HCC) which accounts for 70% of liver cancers (Bray et al. 2024). Recent advances in curative treatments include liver resection, transplantation, and locoregional therapies for early HCC, and for advanced HCC, newer combinations of molecular-targeted agents (MTA) with immune checkpoint blockade (Suzuki et al. 2024). Yet, the 5-year survival remains dismal at 15–20%, which underscores the critical need for improved early detection and risk stratification for HCC.

**Fig. 1:**
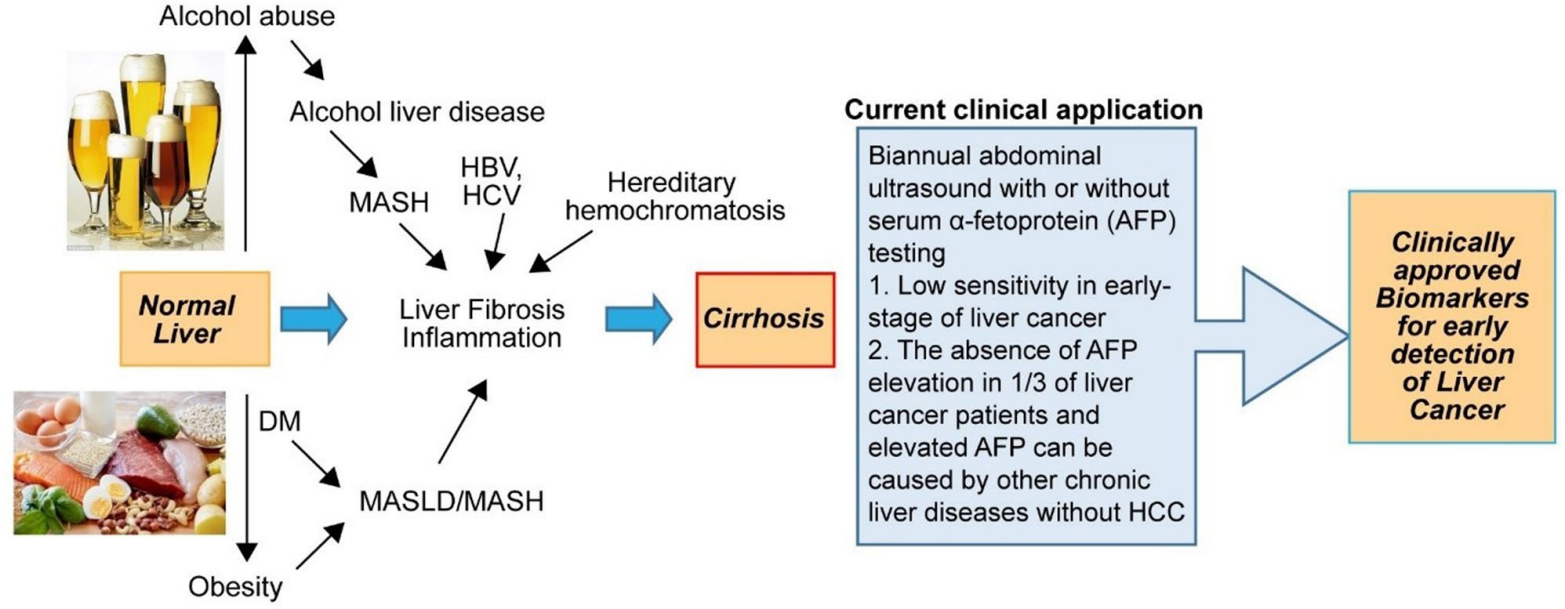
High risk populations and current clinical approaches for HCC surveillance

Major risk factors for HCC include chronic viral hepatitis (HBV and HCV), alcohol use, diabetes, obesity, metabolic dysfunction-associated steatotic liver disease/steatohepatitis (MASLD/MASH) (Konyn et al. 2021; Cho et al. 2023; Qiu et al. 2024), and hereditary disorders such as hemochromatosis (Atkins et al. 2020). These conditions can lead to progressive liver injury characterized by inflammation, necrosis, and regeneration (cirrhosis) (Alberts et al. 2022; Barton et al. 2018; Flemming et al. 2021). Current HCC screening guidelines primarily recommend AFP testing and ultrasound (US) for high-risk patients with chronic HBV infection and/or cirrhosis (Fig. 1). Studies have shown that the combination of AFP and US significantly enhances sensitivity for the detection of early-stage HCC (Tzartzeva et al. 2018). Although biannual screening using US plus AFP has shown promise in HBV patients (Zhang et al. 2004), reducing HCC mortality in this group by 37%, its limitations are compounded by the widespread use of antiviral therapies and the rising prevalence of obesity and MASLD/MASH (Esfeh et al. 2020), for whom screening guidelines are less well-defined.

**Fig. 2:**
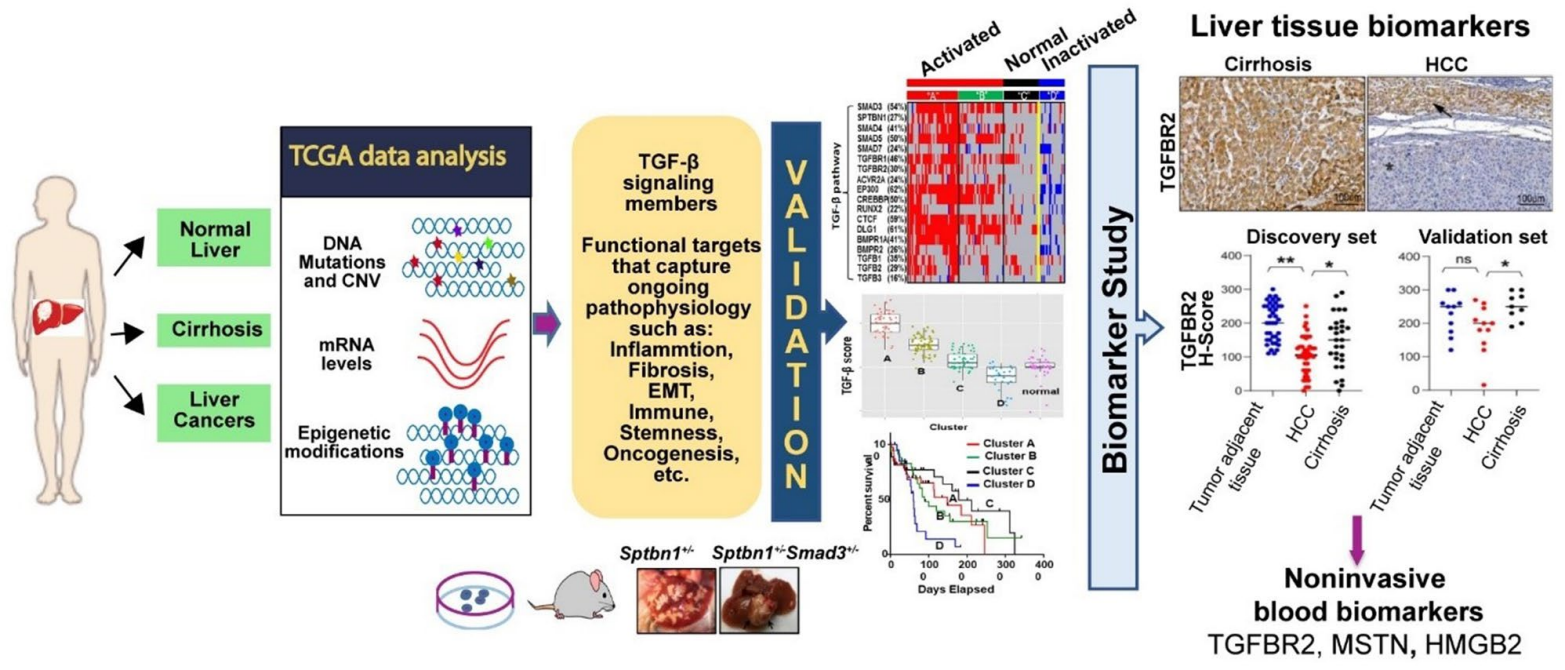
Integrated approaches for functional biomarker studies in HCC that capture ongoing biology in the liver: TGF-β pathway

Challenges in identifying robust markers that stratify risk and predict HCC include molecular heterogeneity, multiple etiologies, and diverse pathology (Fig. 2). The development of chromosomal instability with progressive accumulation of genetic and epigenetic alterations is best understood from large-scale multi-genomic human studies paired with animal models and mechanistic insight into HCC. The human Cancer Genome Atlas (TCGA) characterization of multiple cancer types as well as 363 HCC cases (Chen et al. 2018; Cancer Genome Atlas Research Network 2017; Korkut et al. 2018; Liu et al. 2018; Malta et al. 2018) has given new insight into frequent mutational analyses in multiple pathways. For example, the characterization of 363 HCC cases includes WNT signaling (44%), p53 (31%) and Telomerase (*TERT* promoter mutations in 44%), *CDKN2A* silencing in 53% as well as broader genomic alterations in the TGF-β signaling (43%). Moreover, PI3K, Myc, and Met signaling pathways, among others, play an important role and are described in greater detail in this review, together with animal models.

This review explores recent advances in non-invasive biomarkers for HCC diagnosis from the past five years, focusing on circulating biomarkers (proteins, DNA, and RNA), the gut microbiome, and imaging markers. We highlight biologically functional markers, identified through an integrated approach with animal models, that can stratify HCC risk by reflecting ongoing liver pathology, progression from fatty liver disease to cirrhosis, and ultimately, to cancer. The ideal biomarker should have a sensitivity above 85% and specificity above 95% for risk stratification of disease for cancer (Passaro et al. 2024). Here, we emphasize the incorporation of these functional biomarkers with diagnostic algorithms. By examining recent phase 2–3 clinical trials, we address their potential to stratify risk, improve early HCC detection, and improve patient outcomes.

### Protein biomarkers development with phase 3 evaluation and more

**Alpha-fetoprotein (AFP)** is a glycoprotein implicated in multiple aspects of HCC progression, including roles in hepatocyte proliferation, invasion, metastasis, apoptosis, and immune evasion (Chen et al. 2020; H. I. Kim et al. 2022b). A meta-analysis of 41 studies revealed a suboptimal performance for AFP in detecting early-stage HCC (overall sensitivity 49%, specificity 88%) (Singal et al. 2022), while the combinational use of US with AFP improved the sensitivity (74%) but decreased the specificity (83.9%). AFP levels alone are elevated in only two-thirds of HCC patients, and false positives occur frequently in individuals with other liver conditions, limiting AFP’s standalone utility (Y. T. Lee et al. 2021b). Temporal measurements with progression of disease may enhance early detection accuracy compared to single measurements (Philips et al. 2021). Additionally, HCC patients with MASLD typically had lower AFP levels compared to those with viral HCCs (Than et al. 2017). Given the modest standalone performance of AFP (sensitivity ranging from ~ 30–50% in phase 3 studies), further studies are required to validate its combined use with novel biomarkers across diverse populations to enhance early-stage detection and risk stratification.

**AFP-L3**, lens culinaris agglutinin-reactive fraction of AFP, a liver-specific variant of AFP, differentiates increases in AFP from HCCs as opposed to benign liver disease (Lee et al. 2021a ) and is potentially useful in cases with intermediate AFP levels (20–200 ng/mL) (Sterling et al. 2007). In patients with cirrhosis, comparable diagnostic sensitivity for HCC were observed between AFP-L3 and AFP in two American and one European prospective phase 3 studies (Beudeker et al. 2023; Singal et al. 2022b; Tayob et al. 2023) (Table 1). The improved sensitivity of AFP-L3 over AFP (46.6% vs. 34.5%) observed in a Latin American cohort of patients with cirrhosis suggests its potential for enhanced HCC detection in this population, though further validation is warranted (Beudeker et al. 2023) (Table 1). A meta-analysis of six studies (*n* = 2447) found that AFP-L3 has high specificity (92%) but low sensitivity (34%) for early HCC diagnosis (Zhou et al. 2021). Thus, AFP-L3 may be more useful for ruling out HCC in patients with elevated AFP than for early HCC detection.

**Table 1:** Biomarkers

Biomarker	Study type, No. of subjects, and Biomarker development phase	Sensitivity (%)	Specificity (%)	Cut-off	AUROC	Ref.	Notes*
AFP	Prospective (*n* = 2331), phase 3	38.4	90	8.6 ng/mL	0.72	PMID: 38899967 El-Serag et al., 2024	Cirrhosis any etiology, HCC
AFP	Prospective (*n* = 534), phase 3	34.6–38.2	90	10.8–11.2 ng/mL	0.71–0.78	PMID: 35124267 Tayob et al. 2023	Cirrhosis any etiology, HCC
AFP	Prospective (*n* = 397), transition from phase 2 to 3	50	90	17.4 ng/mL	0.77	PMID: 34618932 Singal et al. 2022b	Cirrhosis any etiology, HCC
AFP	Prospective (*n* = 1084), phase 2	42.4	94.9	20 ng/mL	0.844	PMID: 37938100 Piratvisuth et al. 2023	Chronic liver disease (> 77% viral etiology), HCC
AFP	Retro/prospective (*n* = 437), phase 2	43	98	20 ng/mL	0.81	PMID: 32889146 Chalasani et al. 2021	Liver disease any etiology (> 87.4 cirrhosis) and HCC
AFP	Prospective (*n* = 163), phase 2	75	93.5	14.2 ng/mL	0.869	PMID: 36013482 Hadi et al. 2022	Non-cirrhosis and cirrhosis with etiology (HBV/HCV/MASH), HCC
AFP	Prospective (*n* = 1120), phase 2	44.8	76.1	20 ng/mL	0.692	PMID: 31358576 Cai et al. 2019	HBV, cirrhosis and HCC
AFP	Prospective (*n* = 288), phase 3	29.2	87.4	20 ng/mL	0.59	PMID: 37708457 Beudeker et al. 2023	European cirrhosis and HCC
AFP	Prospective (*n* = 284), phase 3	34.5	92.4	20 ng/mL	0.66	PMID: 37708457 Beudeker et al. 2023	Latin America cirrhosis and HCC
AFP-L3	Prospective (*n* = 2331), phase 3	37.6	90	7.5%	0.61	PMID: 38899967 El-Serag et al., 2024	Cirrhosis any etiology, HCC
AFP-L3	Prospective (*n* = 534), phase 3	34.6–41.2	90	8.3–8.4%	0.64–0.81	PMID: 35124267 Tayob et al. 2023	Cirrhosis any etiology, HCC
AFP-L3	Prospective (*n* = 397), transition from phase 2 to 3	46.2	90	11.9%	0.80	PMID: 34618932 Singal et al. 2022b	Cirrhosis any etiology, HCC
AFP-L3	Prospective (*n* = 288), phase 3	30.4	81.1	10%	0.56	PMID: 37708457 Beudeker et al. 2023	European cirrhosis and HCC
AFP-L3	Prospective (*n* = 284), phase 3	46.6	91.7	10%	0.69	PMID: 37708457 Beudeker et al. 2023	Latin America cirrhosis and HCC
AFP-L3	Retro/prospective (*n* = 437), phase 2	56	93	10%	0.81	PMID: 32889146 Chalasani et al. 2021	Liver disease any etiology (> 87.4 cirrhosis) and HCC
DCP	Prospective (*n* = 2331), phase 3	30.4	90	2.91 ng/mL	0.75	PMID: 38899967 El-Serag et al., 2024	Cirrhosis any etiology, HCC
DCP	Prospective (*n* = 534), phase 3	17.6–23.1	90	1.4–1.5 ng/mL	0.68–0.72	PMID: 35124267 Tayob et al. 2023	Cirrhosis any etiology, HCC
DCP	Prospective (*n* = 288), phase 3	26.7	87.4	7.5 ng/mL	0.57	PMID: 37708457 Beudeker et al. 2023	European cirrhosis and HCC
DCP	Prospective (*n* = 284), phase 3	36.2	97.4	7.5 ng/mL	0.65	PMID: 37708457 Beudeker et al. 2023	Latin America cirrhosis and HCC
DCP	Prospective (*n* = 397), transition from phase 2 to 3	34.6	90	5.9 ng/mL	0.70	PMID: 34618932 Singal et al. 2022b	Cirrhosis any etiology, HCC
DCP	Prospective (*n* = 1084), phase 2	61.3	88.7	20 ng/mL	0.772	PMID: 37938100 Piratvisuth et al. 2023	Chronic liver disease (> 77% viral etiology), HCC
DCP	Retro/prospective (*n* = 437), phase 2	39	93	7.5 ng/mL	0.83	PMID: 32889146 Chalasani et al. 2021	Liver disease any etiology (> 87.4 cirrhosis) and HCC
DCP	Prospective (*n* = 163), phase 2	90	82.1	36.7 mAU/mL	0.905	PMID: 36013482 Hadi et al. 2022	Non-cirrhosis and cirrhosis with etiology (HBV/HCV/MASH), HCC
DCP	Retrospective (*n* = 186), phase 2	72	71	7 ng/mL	0.715	PMID: 38994169 He et al. 2024	Healthy control and AFP-negative HCC
OPN	Retrospective (*n* = 322), phase 2	79.2	79.6	-	0.85	PMID: 32043608 Zhu et al. 2020	Chronic hepatitis, cirrhosis, and HCC
MDK	Meta-analysis of 9 studies	87	86	0.5 ng/mL	0.95	PMID: 31600291 Zhang et al. 2019	HCC
MDK	Meta-analysis of 17 studies	84	82	-	0.89	PMID: 32039435 Lu et al. 2020	HCC
GP73	Retrospective (*n* = 186), phase 2	75.6	93	103 ng/mL	0.843	PMID: 38994169 He et al. 2024	Healthy control and AFP-negative HCC
GPC3	Retrospective (*n* = 344), phase 2	80	85	0.0414 ng/mL	0.88	PMID:32087138 Liu et al. 2020	Healthy control and HCC
TGF-β1	Prospective	53.1%	98.9%	50 ug/ g-1 creatinine	0.730	PMID: 9166938 Tsai et al. 1997	Cirrhosis (74.4% viral etiology), HCC
SCCA-IgM	Prospective	89%	50%	130 AU/mL	0.63	PMID:24635038 Pozzan et al. 2014	Cirrhosis, healthy control, and HCC

*Notes refer to the main groups included in the studies, specifically detailing the composition of the control groups

Des-γ-carboxy prothrombin (**DCP**), also known as Prothrombin Induced by Vitamin K Absence (PIVKA-II), which is significantly elevated in serum of HCC patients (Liebman et al. 1984), has been widely used in Japan and China for HCC diagnosis and surveillance (Kim et al. 2023). Autocrine/paracrine secretion of DCP has been implicated in promoting HCC proliferation and angiogenesis through activation of the JAK/STAT3 and PLCγ/MAPK signaling pathways (Fujikawa et al. 2007; Suzuki et al. 2005). In phase 2 and 3 studies of cirrhotic patients, DCP exhibited lower sensitivity for HCC detection compared to both AFP and AFP-L3 (DCP sensitivity: from 17.6% to 36.2; AFP and AFP-L3 sensitivities: from the 30.4–50%) (Beudeker et al. 2023; El-Serag et al. 2025; Singal et al. 2022b; Tayob et al. 2023) (Table 1). Moreover, diagnostic performance of DCP varies significantly depending on the etiology of liver disease, demonstrating higher sensitivity but lower specificity in patients with viral infections (Hadi et al. 2022; Marrero et al. 2009; Piratvisuth et al. 2023). AFP-L3% with AFP and DCP utilized in GALAD assays enhance HCC detection rates (Chen et al. 2020; Singal et al. 2022b; Tayob et al. 2023) (Table 3).

**Table 2:** Microbiome

Biomarkers	Samples	Sample Size	Findings	Diagnosis/Prognosis Potential	Reference
Gut Microbiome	Fecal	16 healthy controls, 33 patients with viral-HCC (17 and 16 cases with hepatitis B virus (HBV) and hepatitis C virus (HCV) infection, respectively), and 18 patients with NBNC-HCC	*Bacteroides*,* Streptococcus*,* Ruminococcus gnavus group*,* Veillonella*,* and Erysipelatoclostridium* ↑ *Romboutsia*,* UCG-002*,* Lachnospiraceae NK4A-136*,* Eubacterium hallii group*,* Lachnospiraceae ND-3007 group*,* Erysipelotrichaceae UCG-003*,* and Bilophila* ↓	Identify a gut microbiota signature in differentiating between viral-related HCC (Viral-HCC) and non-hepatitis B-, non-hepatitis C-related HCC (NBNC-HCC)	PMID: 38183473 Jinato et al. 2024
Gut Microbiome	Fecal	124 patients diagnosed with HBV-associated HCC and 82 HBV‐related hepatitis, and 86 healthy volunteers	*Dialister*,* Veillonella*,* Eubacterium coprostanoligenes group*,* and Lactobacillus genus* ↑ *Bacteroides* ↓	TNM, AST, *Veillonella*, and *Streptococcus pneumoniae* used in a model for the prognosis/early recurrence of HBV induced HCC, AUROC: 0.78	PMID: 37778742 Zheng et al. 2023
Gut Microbiome	Fecal	30 HCC-cirrhosis patients, 38 cirrhotic patients without HCC, and 27 age- and body mass index [BMI]-matched healthy volunteers	*Veillonella and Scardovia* ↑ *Lachnospira*,* Ruminococcus*,* and Butyricicoccus* ↓	Demonstrates the potential of fecal microbes as tools for noninvasive diagnosis or microbiome-oriented interventions in HCC-cirrhosis	PMID: 32546668 Lapidot et al. 2020
Oral Microbiome	oral	90 HCC cases and 90 controls with oral samples obtained from a larger population-based case-control study of 673 patients with HCC and 1,166 controls	Cyanobacteria positively associated with HCC	Novel evidence that oral Cyanobacteria may be an independent risk factor for HCC	PMID: 34697061 Hernandez et al. 2022

↑: Upregulated in HCC/Early Recurrence ↓: Downregulated in HCC/No Early Recurrence/ Healthy Controls

Sequencing method used for the above studies: 16 S rRNA sequencing

### Protein biomarkers with phase 2 evaluation studies

**Osteopontin (OPN)**, a secreted extracellular matrix protein, that interacts with Integrins, functions as a Th1 cytokine, is involved in tissue remodeling (Lund et al. 2009) and intricately linked to the JAK2/STAT3 and PI3K/Akt signaling pathways in HCC, contributing to tumor growth, invasion, and metastasis (Desert et al. 2022; Wu et al. 2022; Yu et al. 2018). Studies also found that increased secretion of OPN contributed to promoting the synthesis of collagen-I in hepatic stellate cells via inducing HMGB1 (Arriazu et al. 2017), which is involved in chronic liver disease (Song et al. 2021). Increased plasma OPN results are similar to AFP (Abu El Makarem et al. 2011; Jang et al. 2016; Simão et al. 2015). A recent Chinese cohort study with 105 cases of chronic hepatitis, 116 of liver cirrhosis, and 101 of HCC showed that serum OPN analyses gave a better AUROC of 0.851 (79.2% sensitivity and 79.6% specificity) compared with AFP (AUROC of 0.683) or DKK1 (AUROC of 0.639) (Zhu et al. 2020) (Table 1). In AFP-negative samples, serum OPN also performed well with an AUROC of 0.838.

**Midkine (MDK)**, a heparin-binding growth factor, activates multiple key pathways such as MAPK, WNT, and TGF-β, leading to increased cancer proliferation, angiogenesis, and metastasis (Du et al. 2022; Sun et al. 2017). A meta-analysis of 9 studies showed that MDK displayed diagnostic efficacy for HCC with a cutoff value of 0.5 ng/mL: an AUROC of 0.95, sensitivity 87%, and specificity 86%) (Zhang et al. 2019) (Table 1). Another systematic meta-analysis of 17 studies further confirmed that MDK showed better performance in diagnosing early-stage HCC than AFP: AUROC, 0.89 vs. 0.52, sensitivity, 84% vs. 44%, specificity, 82% vs. 85% (Lu et al. 2020) (Table 1). Also, MDK showed promising performance in AFP negative HCC: an AUROC of 0.91, sensitivity 89%, and specificity 84%. A recent study validated the functional role of circulating MDK in promoting liver carcinogenesis via activating PI3K/AKT/mTOR signaling (Du et al. 2022), indicating that MDK is a promising biomarker that deserves further validation.

**Dickkopf-1 (DKK1)** promotes liver cancer invasion and metastasis via β-catenin/MMP7 signaling (Chen et al. 2013). DKK1 genetic deletion impairs HCC cell invasion, proliferation, and tumor development (Seo et al. 2021). Furthermore, analysis of tissue microarray data suggests that DKK1 may serve as a new prognostic predictor for HCC patients, particularly for those with normal AFP levels and those in the early stages of the disease (Yu et al. 2009). In a large-scale multicenter study (*n* = 1284), serum DKK1 levels were significantly elevated in patients with HCC compared to those with cirrhosis or chronic HBV infection(Shen et al. 2012), which displayed complementary diagnostic potential with AFP. For early-stage HCC, DKK1 demonstrated superior diagnostic accuracy (AUROC 0.85 vs. 0.658 for AFP), with higher sensitivity (70.9% vs. 54.4%) and specificity (84.7% vs. 69.3%). Combining DKK1 and AFP further improved performance, achieving 84.9% sensitivity and 77.4% specificity. Similarly, two independent cohorts (*n* = 90 and *n* = 80) demonstrated that combination of serum DKK1 and AFP may enhance HCV related HCC diagnostic accuracy (Eldeeb et al. 2020; Fouad et al. 2016). Furthermore, studies have revealed DKK1 promoter hypermethylation in liver tissue from HCV-infected patients with chronic liver disease and cirrhosis preceding HCC development (Umer et al. 2014). Taken together, these studies suggest that DKK1 as a potent inhibitor of WNT pathway may serve as a valuable biomarker for early detection of virus-induced HCC.

**Golgi protein-73 (GP73)** is a type II Golgi transmembrane protein found significantly elevated in hepatocytes affected by chronic liver diseases and HCC (Gatselis et al. 2020), which acts as a driver oncogene, initiating intra- and intercellular signaling cascades such as JAK2/STAT3 and ER stress that enhance the angiogenesis and aggressiveness and reshape the tumor microenvironment of HCC (Chen et al. 2015; Wei et al. 2019; Ye et al. 2024). Cleavage releases GP73 and renders it a potentially useful serum biomarker (Gatselis et al. 2020). Given its unique expression in liver tissue from HCC patients, targeting GP73 could provide a strategy to inhibit angiogenesis with reduced off-target effects, as well as a tool for HCC detection. GP73 shows high specificity for HCC and may offer additional diagnostic value, particularly for AFP-negative patients (75.6% sensitivity, 93% specificity) (He et al. 2024; Zhang et al. 2023). Unfortunately, a 36-study meta-analysis revealed moderate diagnostic accuracy for GP73 in cirrhotic patients possibly because of increased GP73 levels in both cirrhotic and HCC patients (Zhang et al. 2023).

**Glypican 3 (GPC-3)**, a member of the heparan sulfate proteoglycan family, is another oncofetal protein found elevated in hepatocellular carcinoma cells and serum small extracellular vesicles (Sun et al. 2023). GPC3 signals through WNT members and extracellular signal-regulated kinase (ERK) pathways (Castillo et al. 2016). Its value may lie in AFP-negative HCC patientswhereGPC-3 displays a sensitivity of 54.6% and a specificity of 76% among AFP-negative patients (AFP < 400ug/L) was observed (Li et al. 2013) (Table 1). Combining AFP and GPC3 improved the sensitivity to 88.1%, but the specificity decreased to 82.7% (Liu et al. 2020), potentially as a combination providing the most useful predictors tested so far.

**Angiopoietin-2 (ANG2)**, associated with tumor angiogenesis (Tanaka et al. 1999, 2002), has been shown to outperform AFP in predicting overall survival (OS) in HCC (Llovet et al. 2012). ANG2-blocking antibodies inhibit tumor angiogenesis and metastasis in mice, suggesting its potential role in future therapeutic targeting (Saharinen et al. 2017). Ang-2 levels are associated with advanced HCC, cases with acute renal injury and higher mortality in decompensated cirrhosis, and liver function indicators such as high MELD and Child-Pugh scores, as well as associated with tumor aggressiveness (Ao et al. 2021; Choi et al. 2021).

#### Viral antigens

HBV antigens, such as HBcrAg, represent promising markers due to their direct involvement in liver pathology and carcinogenesis. HBcrAg levels, which are unaffected by nucleotide analog treatment, provide a reliable indicator of viral replication and intrahepatic activity. Higher HBcrAg levels correlate with increased HCC risk, identifying patients with an inactive virus but elevated HCC risk (Chang et al. 2022). In a study with 2666 patients with chronic HBV infection, HBcrAg levels higher than 10KU/ml positively correlated with increased HCC incidence (Tseng et al. 2019).

**Squamous Cell Carcinoma Antigen (SCCA)** and SCCA-IgM complexes have also emerged as potential markers, with SCCA-IgM showing greater sensitivity and specificity in prognosticating HCC response to therapy. Studies suggest lower SCCA-IgM levels in patients responsive to locoregional therapies, supportive of its diagnostic relevance (Guarino et al. 2017; Pozzan et al. 2014).

### DNA/RNA biomarkers

In a recent case-control cohort study with diverse etiologies (*n* = 558) (Campani et al. 2024), whole-exome sequencing analysis demonstrated that plasma circulating tumor DNA (ctDNA) mutation rates in patients with active HCC were significantly higher (40.2%), compared to that of chronic liver disease control group (1.8%). Consistent with the genomic analysis of liver tissues from TCGA HCC cohort (Cancer Genome Atlas Research Network 2017), the top 5 highest mutations occur in TERT promoter (27.5%), TP53 (21.3%), CTNNB1 (13.1%), PIK3CA (0.2%), and NFE2L2 (0.2%), suggesting these ctDNA mutations may serve as promising non-invasive markers for HCC diagnosis. Another study (*n* = 609) reported that urine ctDNA biomarkers (TP53, RASSF1a, and GSTP1) combined with serum AFP significantly increased the sensitivity for early-stage HCC detection from 62 to 92% (BCLC stage 0, Kim et al. 2022a).

Global 5-hydroxymethylcytosines (5hmC) contents were significantly decreased in liver tissues from patients with early-stage HCC (Liu et al. 2019), which was associated with HBV infection and decreased translocation enzyme activity. As potential effective epigenomic biomarkers, a 32-gene panel that captures 5hmc signature in cell free DNA (cfDNA) significantly discerned early-stage HCC from non-HCC (AUROC of 0.884) and from a high-risk group with chronic hepatitis B virus infection or liver cirrhosis (AUROC of 0.846) in a cohort of 1204 HCC patients and 1350 controls (chronic liver disease and healthy individuals) (Cai et al. 2019) (Table 3). Another independent study (*n* = 262) (Cai et al. 2021) expanding the panel to 64-gene 5hmC signatures in cfDNA further increased the performance for HCC diagnosis (AUROC of 0.93). These studies supported that 5hmC markers could serve as a noninvasive tool for early-stage HCC detection among high-risk subjects.

The better performance of plasma methylated DNA markers (MDMs) for HCC diagnosis (Kisiel et al. 2019) has been validated in a phase 2 study (*n* = 244, AUROC 0.96, sensitivity 95%, specificity 92%), which captures 6-marker changes (HOXA1, EMX1, AK055957, ECE1, PFKP, and CLEC11A normalized by B3GALT6). Recently, in a clinical trial study (NCT03628651), a multiple-target blood-based panel (Chalasani et al. 2021) that combines 4 methylated DNA markers (HOXA1, EMX1, TSPYL5, and B3GALT6) and 2 protein markers (AFP and AFP-L3) outperformed the GALAD score for early-stage diagnosis (AUROC: 0.88 vs. 0.81; sensitivity: 74% vs. 60%, specificity: 90% vs. 86%) (Table 3). The performance of this panel was comparable in patients with virus or non-virus etiologies, and with or without cirrhosis. Moreover, validation of the multi-target panel (HOXA1, TSPYL5, plus AFP and sex) (Chalasani et al. 2022) Using an independent cohort of 156 HCC cases and 245 controls, the multiple target panel that combines methylated DNA markers and protein markers displayed similar performance (AUROC 0.86, sensitivity 72%, specificity 88%) for early-stage HCC diagnosis. These data implied that the multiple target panel may significantly improve early-stage HCC diagnosis.

Several studies highlight circulating microRNAs (miRNA) and exosomal miRNAs could serve as non-invasive biomarkers for HCC surveillance. A serum miRNAs panel that includes six targets (miR-21, miR-221, miR-801, miR-1246, miR-26a, and miR-122) displayed clinical value for the early diagnosis of HCC (AUROC of 0.95) (Zhang et al. 2025). More recently, exosomal miRNAs (miR-10b-5p, miR-221-3p, miR-223-3p, miR-21-5p) may effectively distinguish HCC patients from CH/LC control with AUROC of 0.86, sensitivity of 74%, and specificity of 86% (Ghosh et al. 2020). Another independent study reported a similar performance of a panel including five circulating exosomal miRNAs (miR19-3p, miR16-5p, miR30d-5p, miR-451a, miR-223-3p) with AUROC of 0.85, for distinguishing HCC with non-virus etiology and non-HCC control (Boonkaew et al. 2023).

### Molecular pathways with new insight from animal models

In an ideal situation, a single simple model that replicates the spectrum of HCC from cirrhosis should provide rapid new insight into biologically relevant markers that could stratify risk for HCC. These new insights can be provided from animal models. While no single animal model replicates HCC progression, commonly used preclinical models for HCC include cell lines, organoids, patient-derived xenografts, scaffold-based models, those induced by chemotoxic agents, special diets, genetic modifications, and tumor cell transplantation (He et al. 2015; Zabransky et al. 2023). c-MYC which is overexpressed in up to 70% of viral and alcohol-related human HCCs (Schlaeger et al. 2008) lends itself as a strong GEM model, in which dual (albumin-driven) AEG-1 and Myc overexpression- mice develop aggressive HCCs and lung metastases (Srivastava et al. 2015). GEM models expressing an activated form of β-catenin, the downstream effector of the Wnt pathway, or harboring a liver-specific Apc knockout (KO) showed hepatomegaly or HCC after a long latency (Colnot et al. 2004). In liver-specific p53 KO model through Cre–Lox recombination, the AlfpCre^+^Trp53^Δ2–10/Δ2–10^ mice develop liver cancer in 14 months (Katz et al. 2012). A liver-specific Setd2 depletion model, finding that Setd2 deficiency is sufficient to trigger spontaneous HCC formation (Li et al. 2021). c-MET levels are raised in 20–48% of human HCC samples and represents a potentially therapeutic target (Adebayo Michael et al. 2019; Tao et al. 2017; Venepalli and Goff 2013; You et al. 2011). With Pten expression being reduced in up to 50% and activated mutant forms of PIK3CA in 4% of hepatocellular tumors, liver-specific knockout of Pten in mice develop steatosis and late-onset liver cancer (Horie et al. 2004). Those models provide new insight into biologically relevant biomarkers for HCC.

### Proteins reflecting liver pathophysiology: findings on TGF-β pathway modulation

Animal models combined with analyses of human genomics could ideally provide the most relevant biologically functional biomarkers (Dhanasekaran et al. 2025). For instance, a genomic, epigenomic, and transcriptomic landscape of 44 TGF-β pathway genes and 50 downstream target genes of the pathway in 9,125 patients across all 33 TCGA PanCancer Atlas tumor types (Korkut et al. 2018) revealed that 40% of the cancers carry TGF-β-Smad pathway gene alterations with a common transcription signature; the genomic alterations affect expression of metastatic and epidermal-mesenchymal-transition (EMT) genes; the pathway is most frequently aberrant in Liver and GI cancers, which exhibited 113 of the 176 hotspot mutations identified in the overall cohort.

In cancer, the TGF-β pathway plays apparently contradictory roles, either suppressing (early) or (later) promoting tumor growth. Mouse models of hepatocellular cancers indicate a primarily an early tumor-suppressive role (Chen et al. 2016; David et al. 2016; Katz et al. 2016). Examples include mouse models of HCC with haploinsufficiency of *Tgfbr2*,* Tgfb*, and intercrosses between *Smad3/4* with the adaptor *Sptbn1 (Tgfbr2*^-/-^, *Smad4*^+/-^*Sptbn1*^+/-^ and *Smad3*^+/-^*Sptbn1*^+/-^​ on a C57BL/6 background) (Biswas et al. 2004; Gough et al. 2021a; Gu et al. 2020; Z. Wang et al. 2021b). More recently, we have uncovered obesity-driven HCC in our mouse models with disruption of TGF-β signaling and loss of aldehyde dehydrogenase 2 (Aldh2) (Rao et al., 2021; Yang et al. 2024). ALDH2 detoxifies cells of lipid end products-reactive aldehydes such as 4-HNE that accumulate with a high-fat diet, and the *Aldh2*^*-/-*^*Sptbn1*^*+/-*^ mice provided new insight into the role of obesity in promoting HCC (Yang et al. 2024).

Taking this further, elevated TGF-β1 levels in human HCC tissue are associated with poor prognosis and immune suppression, marking it as a potential target for immunotherapy (Gough et al. 2021; Jin et al. 2022). Both plasma and urine TGF-β1 levels are higher in patients with HCC than in those with cirrhosis, display comparable diagnostic ability as AFP to discriminate HCC from cirrhosis (Song et al. 2002; Tsai et al. 1997). **TGFBR2** is a transmembrane protein that plays a crucial role in regulating TGF-β signaling, which is closely associated with the progression of liver cirrhosis and HCC. Reduced TGFBR2 levels have been observed in liver tissue from HCV-HCC compared with HCV-related cirrhosis patients and healthy subjects, which were significantly correlated with aggressive features of HCC (Abu El-Makarem et al. 2022). A multi-cohort study demonstrated a significant reduction in serum TGFBR2 levels in HCCs compared to cirrhotic liver tissues (Zaidi et al. 2022). Also, circRNA-TGFBR2 has been observed to promote HCC progression via regulating autophagy (Wang et al. 2023), implying a role in risk stratification of HCC. Thus, additional studies are necessary to investigate the potential biomarker value of circulating TGFBR2 in HCC.

**Myostatin (MSTN**,** or GDF8)** is a member of the transforming growth factor beta (TGF-β) superfamily and may prove to be a promising biologically relevant marker, in part from its role as an autocrine inhibitor of muscle growth, contributing to muscle wasting in patients with sarcopenia, which are major issues in cirrhosis and HCC. Sarcopenia is prevalent in up to 40% of cirrhotic patients, particularly with alcoholic liver disease or Child-Pugh class C, linked to a high risk of mortality (Cui et al. 2023; Tantai et al. 2022). Recent studies have identified a causal relationship between sarcopenia and increasing risk of HCC in European populations (Cao et al. 2024), implying that MSTN, the primary mediator of sarcopenia, is a promising biomarker candidate for HCC risk stratification. Consistent with this, a multicenter prospective study found a two-fold increase in serum myostatin levels significantly predicted a higher risk of HCC development in patients with alcoholic cirrhosis (Kim et al. 2020). However, lower MSTN levels were observed in patients with acute decompensation and acute-on-chronic liver failure (ACLF) (Ruiz-Margáin et al. 2023). Therefore, multiple timepoint assays or longitudinal studies are necessary for predicting HCC risk and stratifying the value of MSTN.

**IL-6** is a proinflammatory cytokine that is significantly elevated in both liver tissue and serum of patients with HCC (Kao et al. 2015), specifically in progressive sarcopenia and advanced HCC stage (Choi et al. 2020; Myojin et al., 2022). A systematic meta-analysis of 18 studies demonstrated higher IL-6 levels in HCC patients compared with hepatitis and cirrhosis patients and healthy controls (Shakiba et al. 2018). Serum high mobility group box 1 protein (HMGB1) is a proinflammatory molecule that induces inflammatory cytokine production of TNF-α and IL-6 (Chen et al. 2022; Tripathi et al. 2019). Elevated HMGB1 levels in HCC liver tissue (Liu et al. 2012), are associated with poor prognosis HCC. High mobility group box 2 (HMGB2), closely related to HMGB1 is overexpressed in HCC cells (Kwon et al. 2010; Lu et al. 2023), via activating signaling pathways such as ERK, PI3K/AKT, and Wnt/β-catenin. HMGB2 is involved in stellate cell activation, and serum HMGB2 levels are increased in patients with liver fibrosis and cirrhosis (Huang et al. 2023). Elevated HMGB2 is associated with poor prognosis of HCC patients. Collagen type I α1 (COL1A1) is often overexpressed in cancers, influencing cell proliferation, metastasis, apoptosis, and cisplatin resistance, with high levels linked to poor patient prognosis (Li et al. 2022). COL1A1 is implicated in epithelial-to-mesenchymal transition (EMT) and stemness in HCC (Ding et al. 2024; Ma et al. 2019). COL1A1 levels are higher in HBV-positive cirrhosis and HCC (Mohamed et al. 2021), reflecting a potential for risk stratification of HCC risk in Hepatitis B virus infected patients.

### Gut Microbiome

The liver-gut axis plays a crucial role in liver disease progression and carcinogenesis. Recent studies indicate that specific gut bacteria such as *Bacteroides*, *Streptococcus*, and *Veillonella* are enriched in patients with HCC, especially in non-viral HCC, and may serve as potential biomarkers (Jinato et al. 2024) (Table 2). The presence of viable bacteria within liver tissue further indicates the contributive roles of the gut microbiome in HCC pathophysiology, potentially opening new avenues for non-invasive diagnosis and therapeutic intervention (Huang et al. 2022). Circulating microbial signatures are another emerging subject in the cancer field and are thought to be partially derived directly from the gut via bacterial translocation (You et al. 2022). Similarly, oral Cyanobacteria may be independently associated with HCC risk, possibly via direct impact on the tumor-promoting effects of microcystins and other hepatotoxins and their disruptive influence on lipid metabolism. A 2021 study identified oral Cyanobacteria as an independent risk factor for HCC through bacterial 16 S rRNA sequences in oral samples from 90 HCC cases and 90 controls -part of a larger U.S. case-control study of HCC among patients diagnosed from 2011 to 2016 (Hernandez et al. 2022; Song et al. 2023) (Table 2). Elevated levels of gut bacteria such as *Dialister*,* Veillonella*, and *Eubacterium*, along with their associated metabolites, have been linked to early HCC recurrence (Zheng et al. 2023). Furthermore, increased abundance of *Veillonella* has shown potential for differentiating HCC from cirrhosis (Lapidot et al. 2020). We have observed altered microbiomes in our mouse models with disruption of TGF-β signaling that develop spontaneous HCC and other gastrointestinal cancers (Gu et al. 2020; Z. Wang et al. 2021b). Interestingly, our group and others have observed that these mutant mice do not develop cancers in a germ-free environment (Gu et al. 2020; Maggio-Price et al. 2006). As a novel potential diagnostic tool for HCC, even though the performance of gut microbiome is currently limited in scale and lacking extensive sample validation, the predictive model using gut microbiome together with AFP demonstrated better accuracy (AUROC: 0.9811 vs. 0.8505) (Yang et al. 2023), suggesting its potential complementary effect to the serum testable markers. Future studies should provide new insights into the role of the microbiome in the setting of altered mutational profiles in HCC.

**Table 3:** Biomarker panel and algorithms

Biomarker panel	Study type, No. of subjects, and Biomarker development phase	Sensitivity (%)	Specificity (%)	Cut-off	AUROC	Ref.	Notes*
GALAD	Prospective (*n* = 534), phase 3	30.0-32.4	90	(-0.03)-0	0.75–0.79	PMID: 35124267 Tayob et al. 2023	Cirrhosis any etiology, HCC
GAAD (combing age, sex, AFP, DCP)	Prospective (*n* = 1084), phase 2	71.8	90	2.57	0.907	PMID: 37938100 Piratvisuth et al. 2023	Chronic liver disease (> 77% viral etiology), HCC
GALAD, Single-timepoint	Prospective (*n* = 397), transition from phase 2 to 3	53.8	90	-0.33	0.78	PMID: 34618932 Singal et al. 2022b	Cirrhosis any etiology, HCC
GALAD, longitudinal	Prospective (*n* = 397), transition from phase 2 to 3	69.2	90	-0.33	0.83	PMID: 34618932 Singal et al. 2022b	Cirrhosis any etiology, HCC
GALAD	Prospective (*n* = 288), phase 3	65.8	71.7	-0.63	0.69	PMID: 37708457 Beudeker et al. 2023	European cirrhosis and HCC
GALAD	Prospective (*n* = 284), phase 3	69.8	82.9	-0.63	0.76	PMID: 37708457 Beudeker et al. 2023	Latin America cirrhosis and HCC
GALAD	Prospective (*n* = 2331), phase 3	40	90	-0.38	0.76	PMID: 38899967 El-Serag et al., 2024	Cirrhosis any etiology, HCC
GALAD	Retro/prospective (*n* = 437), phase 2	72	86	-0.63	0.87	PMID: 32889146 Chalasani et al. 2021	Liver disease any etiology (> 87.4 cirrhosis) and HCC
Doylestown	Prospective (*n* = 120)	50	90	0.5	0.98	PMID: 30169533 Mehta et al. 2018	Cirrhosis with any etiology
HES	Prospective (*n* = 397), transition from phase 2 to 3	34.6	90	10.05	0.71	PMID: 34618932 Singal et al. 2022b	Cirrhosis any etiology, HCC
HES	Prospective (*n* = 534), phase 3	36.7–41.2	90	7.94–8.03	0.76–0.82	PMID: 35124267 Tayob et al. 2023	Cirrhosis any etiology, HCC
HES V2.0	Prospective (*n* = 2331), phase 3	47.2	90	1.27	0.77	PMID: 38899967 El-Serag et al., 2024	Cirrhosis any etiology, HCC
5hmc markers (wd-score)	Prospective (*n* = 1120), phase 2	82.7	67.4	27.9	0.846	PMID: 31358576 Cai et al. 2019	HBV, cirrhosis and HCC
Multi-target panel (methylated DNA plus protein)	Retro/prospective (*n* = 437), phase 2	80	90	67	0.92	PMID: 32889146 Chalasani et al. 2021	Liver disease any etiology (> 87.4 cirrhosis) and HCC

*Notes refer to the main groups included in the studies, specifically detailing the composition of the control groups

### Diagnostic algorithms

#### GALAD score

The GALAD score was developed in 2015 and incorporates Gender, Age, and three biomarkers: AFP, AFP-L3%, and DCP to improve the detection of HCC, specifically in patients with chronic liver disease. In phase 2 studies, the GALAD score shows promising results (Table 3). In a cohort of patients with chronic liver disease (*n* = 1084), primarily viral in etiology, the score achieved a high sensitivity of 71.8%, specificity of 90%, and an AUROC of 0.907, underscoring its effectiveness in this group (Piratvisuth et al. 2023). A second phase 2 retro/prospective study with 437 patients demonstrated an AUROC of 0.87, further supporting the diagnostic utility of the GALAD score in a population with a high prevalence of cirrhosis (Chalasani et al. 2021). In a large, phase 3 prospective cohort (*n* = 2331) with cirrhosis of any etiology, the GALAD score exhibited a sensitivity of 40% and specificity of 90%, with an AUROC of 0.76 (El-Serag et al., 2024). A prospective transition study between phase 2 and phase 3 with a cohort of 397 cirrhotic patients showed the advantage of longitudinal assessments of GALAD over single-timepoint scores for HCC diagnosis, with AUROCs of 0.83 and 0.78, respectively, further underscoring the added benefit of longitudinal monitoring (Singal et al. 2022b). While AFP-L3 has been found contributed negligibly in GALAD (Hou et al. 2025; Johnson et al. 2014), two phase 2 prospective studies (*n* = 1006 and *n* = 1142) found that the GAAD score (combining sex, age, AFP, DCP) performed as sensitive (90% and 93.7%) and specific (85.3% and 83.1%) as the GALAD algorithm (sensitivity 93%, specificity 83.3%) in differentiating HCC from chronic liver disease (Hou et al. 2025; Piratvisuth et al. 2023).

### Doylestown algorithm

The Doylestown algorithm incorporates AFP and other laboratory markers (age, gender, alkaline phosphatase and alanine aminotransferase levels) and has demonstrated enhanced specificity and sensitivity over AFP alone (Mehta et al. 2018; Wang et al. 2016) (Table 3), especially at early detection time points. Further development may enhance its utility, particularly in racial and ethnic minority populations where HCC disparities persist. In a prospective study involving 120 patients with cirrhosis of various etiologies, the Doylestown algorithm achieved a sensitivity of 50% and a specificity of 90%, with an AUROC of 0.98 (Mehta et al. 2018) (Table 3). This high AUROC suggests that the Doylestown algorithm may outperform traditional single biomarker approaches, particularly in early HCC detection, where sensitivity and specificity are critical.

### Hepatocellular carcinoma early detection screening

Hepatocellular Carcinoma Early Detection Screening (HES) is a screening technique that combines AFP with age alanine aminotransferase and platelets. HES has been evaluated across multiple cohorts to assess its diagnostic effectiveness in detecting HCC among patients with cirrhosis (Table 3). In a phase 2–3 transition study involving 397 patients with cirrhosis of varying etiologies, the HES score demonstrated a sensitivity of 34.6% with a specificity of 90%, yielding an AUROC of 0.71, highlighting its moderate diagnostic accuracy in this group (Singal et al. 2022). In a larger phase 3 cohort of 534 patients, the HES score showed improved sensitivity, ranging from 36.7 to 41.2%, with a consistently high specificity of 90% and AUROC values between 0.76 and 0.82, indicating enhanced performance in a broader population (Tayob et al. 2023).

The updated HES v2.0, which incorporates AFP-L3 and DCP in addition to the original HES components, was evaluated in a large phase 3 prospective cohort of 2,331 patients with cirrhosis. This newer version showed an increased sensitivity of 47.2% while maintaining the specificity at 90% and achieving an AUROC of 0.77 (El-Serag et al., 2024). These results suggest that HES v2 provides a modest improvement over the original HES score in detecting HCC, especially in more extensive and diverse patient populations with cirrhosis, potentially enhancing its utility in clinical practice for early HCC detection.

### Imaging markers

#### MRI

Magnetic Resonance Imaging (MRI) is highly effective for detecting hepatocellular carcinoma (HCC). A meta-analysis of 15 studies involving 2,807 patients showed that MRI demonstrated high diagnostic accuracy with a pooled per-patient sensitivity of 86% and specificity of 94%, while per-lesion sensitivity was 77%. This diagnostic performance was consistent across different MRI protocols, both with and without contrast enhancement, and was superior to ultrasound, which had a sensitivity of 53% (Gupta et al. 2021). Similar findings were reported from another study involving 22 studies and 1685 patients, mentioning that multi-sequence non-contrast MRI (NC-MRI) achieved a pooled per-patient sensitivity of 86.8% and specificity of 90.3%. NC-MRI also maintained high sensitivity for detecting smaller lesions (< 2 cm) at 77.1%, compared to 88.5% for lesions > 2 cm (Chan et al. 2022). The application of deep learning to interpret MRI images is rapidly advancing, achieving high diagnostic performance and potentially aiding less experienced radiologists in early HCC detection.

### Vibration-controlled transient elastography (VCTE)

Liver stiffness measurement (LSM) by vibration-controlled transient elastography (VCTE, FibroScan) which is a non-invasive diagnostic biomarker of liver fibrosis, is a promising method for HCC risk stratification in cirrhosis. Recently, a retrospective study (*n* = 1850) reported that the HCC risk in HCV cirrhotic patients after a sustained virological response (SVR) was positively correlated with the increase of LSM, especially for those patients with LSM above 10 kPa (John et al. 2024). Another Swedish multi-center cohort study (*n* = 14414) further supported that increased LSM was significantly associated with increased HCC risk for patients with cirrhosis and chronic liver diseases across different etiologies (Hegmar et al. 2024). An LSM-based machine learning algorithm displayed superior performance for stratifying 5-year HCC risk among patients with chronic liver disease (AUROC of 0.89), which was separately validated in the Hong Kong (*n* = 2732) and Europe (*n* = 2384) cohorts, and was significantly better than other existing HCC risk scores such as aMAP score, Toronto HCC risk index, and 7 hepatitis B-related risk scores (Lin et al. 2024).

### AI-enhanced imaging and biomarker integration

Artificial intelligence (AI)-enhanced CT scans improve diagnostic accuracy, achieving sensitivities up to 89.4% in complex cases. AI-assisted imaging holds the potential for automated HCC detection, particularly for early-stage disease, thus supporting more timely and accurate diagnosis. A deep-learning AI system trained on CT images from 7,512 patients, validated and achieved an area under the receiver-operating characteristic curve (AUROC) of 0.887 and 0.883 for the internal and external test sets, respectively. The AI system demonstrated high accuracy (81.0% for the internal test set and 81.3% for the external test set) and high sensitivity (78.4% for the internal test set and 89.4% for the external test set) (Wang et al. 2021a ).

AI-based analysis has also refined our tissue-based assessment of TGFBR2 in cirrhotic versus HCC tissue samples, demonstrating reduced TGFBR2 levels as a promising biomarker for HCC detection (Zaidi et al. 2022). Our AI-enhanced model improved accuracy (sensitivity of 0.7, specificity of 0.54) and revealed a reduction in TGFBR2 in HCC compared to cirrhotic tissue, highlighting its potential as a diagnostic tool.

### Cost-effectiveness of biomarker-based screening

Several studies and models suggest HCC surveillance using magnetic resonance imaging (MRI) and/or ultrasound is cost-effective in patients with compensated cirrhosis (Goossens et al. 2017; Nahon et al. 2022), particularly when considering quality-adjusted life years (QALY) gained. Latest cost-effectiveness analysis supports the potential viability of future biomarker-based HCC screening (Singal et al. 2024). Both ultrasound/AFP and biomarker-based screening strategies are cost-effective compared to no screening at a willingness-to-pay threshold of $150,000/QALY. However, biomarker-based screening demonstrated a lower incremental cost-effectiveness ratio and has been favored in a greater proportion of simulations. Sensitivity analyses reveal that screening adherence, costs, and sensitivity for early-stage HCC detection influenced the cost-effectiveness of the evaluated strategies. The cost of screening per quality-adjusted life years decreases with increasing HCC risk, making it crucial to accurately stratify patients and consider factors like etiology and disease stage when making screening decisions.

### Limitations in current research

Several methodological challenges exist across HCC biomarker studies. Many studies rely on derivation samples without independent validation, leading to optimistic AUROC estimates that may not generalize to broader populations. Additionally, multi-center studies often fail to account for center effects, which can introduce confounding variables. Model calibration and handling of outliers are also inconsistently reported, potentially skewing diagnostic accuracy. Combinatorial strategies for the surveillance and diagnosis of HCC, exemplified by the GALAD and HES algorithms, which incorporate AFP alongside additional biomarkers, have shown considerable promise following rigorous phase 3 prospective validations. Nonetheless, the multitude of variables—including protein markers, clinical characteristics, and varied analytical methodologies—can lead to the development of predictive models that may demonstrate comparable performance metrics but differ significantly in their applicability across distinct geographical regions, etiological contexts, and stages of disease. While the GALAD and HES biomarker panel demonstrate improved HCC detection sensitivity in patients with chronic liver disease (Piratvisuth et al. 2023; Chalasani et al. 2021), their performance (sensitivity, specificity, and AUROC) decreases substantially in high-risk cirrhotic cohorts (Tayob et al. 2023; EI-Serag et al., 2024; Beudeker et al. 2023), implying the need for further refinement and validation in specific high-risk populations. Furthermore, ongoing biomarker studies under phase 3 face similar challenges, such as incomplete cohort data, potential selection bias during sample acquisition, and limitations in sample sizes for both discovery and validation cohorts. The inherent heterogeneity of HCC, encompassing a wide range of underlying etiologies and risk factors, further complicates the quest for a single, universally applicable biomarker. This complexity highlights the imperative for a comprehensive approach to HCC diagnosis and monitoring, one that integrates a variety of biomarkers, particularly those with biological relevance, and clinical data to enhance the specificity and sensitivity of detection across diverse patient populations. Adherence to rigorous epidemiological standards, such as STROBE guidelines, could improve the reliability and applicability of future research. It should be noted that the final effectiveness of those potential HCC surveillance strategy will be further challenged as the US-based screening when applicated in resources-limited settings (Parikh et al. 2023), such as lacking experienced health providers, varied image visualization and biomarker test performance, and with patients lacking up-to-date knowledge and low adherence.

## Conclusions

The landscape of HCC biomarkers is evolving, driven by advances in molecular biology, genomics, and AI-enhanced imaging. While traditional biomarkers like AFP and DCP remain valuable, new candidates from the fields of circulating DNA, the gut microbiome, and diagnostic algorithms hold the promise of improved sensitivity and specificity. The molecular heterogeneity and complex signaling pathways underlying HCC present both challenges and opportunities in biomarker development. Advances in research in pathways such as the TGF-β members, together with animal models, have provided valuable insights into HCC pathogenesis, paving the way for biological biomarker strategies (Fig. 2). Our ongoing work in serum proteomics, informed by TGF-β pathway components, provides a new foundation for novel predictive models that enhance risk stratification of HCC patients. Integrating multi-modal data encompassing proteomic and imaging biomarkers with established clinical parameters, coupled with advanced AI-driven analytical approaches, offers a promising avenue for refining risk stratification algorithms and ultimately improving patient outcomes. Future research efforts should prioritize the validation of these biomarkers across large-scale prospective studies assessing their diagnostic performance in cohorts with diverse populations and etiologies. Ultimately, these findings should be translated into precision HCC surveillance and therapeutic strategies tailored to individual risk profiles. Robust collaborations across institutions and industries will be critical to advancing these biomarkers from the bench to the clinic.

## Data Availability

No datasets were generated or analysed during the current study.

## Abbreviations

HCC: Hepatocellular carcinoma
AFP: α-fetoprotein
US: Ultrasound
MASLD/MASH: Metabolic dysfunction-associated steatotic liver disease/steatohepatitis
HBV: Hepatitis B
HCV: Hepatitis C
MAPK: Mitogen-activated protein kinase
PI3K: Phosphoinositide 3-kinase
TGF-β: Transforming growth factorβ
TCGA: The human Cancer Genome Atlas
DCP: Des-γ-carboxy prothrombin
GP73: Golgi protein-73
GPC3: Glypican-3
ERK: Extracellular signal-regulated kinase
ANG2: Angiopoietin-2
SCCA: Squamous Cell Carcinoma Antigen
ctDNA: Circulating tumor DNA
COSMIC: Somatic Mutations in Cancer
MSTN: Myostatin
ACLF: Acute-on chronic liver failure
HMGB1: Serum high mobility group box 1 protein
HMGB2: High mobility group box 2
COL1A1: Collagen type I α1
EMT: Epithelial-to-mesenchymal transition
5-hmC: 5-Hydroxymethylcytosine
MDM: Methylated DNA marker
cfDNA: Cell-free DNA
BCLC: Barcelona Clinic Liver Cancer
NC-MRI: Non-contrast MRI
VCTE: Vibration-controlled transient elastography
LSM: Liver stiffness measurement
AI: Artificial intelligence
